# Perceptions of nicotine harm among adults who use little cigars and cigarillos: A cross-sectional analysis of wave 7 of the Population Assessment of Tobacco and Health (PATH) Study 2022–2023

**DOI:** 10.18332/tid/214722

**Published:** 2026-01-16

**Authors:** Amanda Fidalgo, Michael J. Halenar, Brittany Merson, Apoorva O. Rajan-Sharma

**Affiliations:** 1 Center for Tobacco Products, Food and Drug Administration, Silver Spring, United States

**Keywords:** cigarillos, cigarette, little cigars, nicotine perceptions, PATH

## Abstract

**INTRODUCTION:**

Little is known about nicotine perceptions among people who use little cigars and cigarillos (LCCs). Nicotine perceptions may influence how people respond to changes in the tobacco marketplace, including changes that would result from regulatory actions such as a proposed nicotine product standard. This study examines differences in nicotine harm misperceptions between adults who use LCCs, those that use cigarettes, and those that use both products.

**METHODS:**

We conducted a cross-sectional analysis of a nationally representative sample of US adults (aged ≥18 years) that use LCCs and/or cigarettes (n=5507) from the Population Assessment of Tobacco and Health (PATH) Study Wave 7 (2022–2023). We estimated the percentage of people who used LCCs that overestimated (perceived nicotine as ‘very’ or ‘extremely’ harmful) or were incorrect (either overestimate the harm or perceive nicotine as ‘not at all’ harmful) about nicotine harms and compared this to those who exclusively used cigarettes and those who dual used both products.

**RESULTS:**

Approximately 63.0% of respondents who use LCCs overestimated nicotine harms and 65.7% reported nicotine misperceptions. We found no significant difference in nicotine harm perceptions between people who exclusively used LCCs (overestimation, adjusted odds ratio AOR=1.05; 95% CI: 0.82–1.34; incorrect AOR=1.19; 95% CI: 0.93–1.53), exclusively use cigarettes (base category), and used both products (overestimation AOR=1.05; 95% CI:0.80–1.39; incorrect AOR=1.16; 95% CI: 0.88–1.53). People who used other tobacco products in addition to LCCs and/or cigarettes were significantly less likely (overestimation AOR=0.70; 95% CI: 0.61–0.81; incorrect AOR=0.73; 95% CI: 0.63–0.84) to overestimate the harms of nicotine compared to those who did not use other tobacco products.

**CONCLUSIONS:**

People who use LCCs are equally likely to overestimate or be incorrect about nicotine harms as those who exclusively or dual use cigarettes, but using additional products is associated with correct responses about nicotine harms.

## INTRODUCTION

The FDA has regulatory authority to set nicotine levels for tobacco products in the US^1^. There is evidence that consumer harm perceptions of nicotine are associated with tobacco product use^2,3^. Existing research indicates that over half of all people who use cigarettes incorrectly believe nicotine is extremely harmful to health or the primary driver of smoking-related cancers^4,5^. Limited research addresses nicotine perceptions of people who use other combustible tobacco products. Cigars are the second most popular combustible tobacco product, following cigarettes, among US adults^6^. Within the cigar category, little cigars and cigarillos (LCCs) are the most popular in the US, with approximately 1.5 and 2.8 million people using little cigars and cigarillos, respectively^7^. To our knowledge, there are no nationally representative estimates of the prevalence of nicotine harm misperceptions among adults who use LCCs, and the only published data in this area come from qualitative analyses^8,9^.

This project uses nationally representative data from the Population Assessment of Tobacco and Health (PATH) Study Wave 7 (W7; 2022–2023) to evaluate nicotine harm perceptions among adults who use LCCs. Because dual use of cigarettes and LCCs is common^7^, this project also compares the nicotine harm perceptions of adults who use LCCs without cigarettes, cigarettes without LCCs, and both LCCs and cigarettes.

## METHODS

### Study design

The PATH Study is an ongoing, nationally representative, longitudinal cohort study conducted in the US^10,11^. The current study analyzed adult (aged ≥18 years) data from the Wave 7 (W7) cohort in the W7 Restricted-Use File (RUF)^12^ (2022–2023; n=29780). We examined adults who currently use LCCs and/or cigarettes (n=5507). Cross-sectional full-sample and replicate weights were created for the W7 cohort to adjust for the complex sample design and non-response. The weights allow for statistically valid estimates representing the civilian non-institutionalized population of the US aged ≥18 years at W7. The replicate weights enable computation of associated measures of statistical precision. Further details about the PATH W7 Study design, methods, and reliability and validity of responses are published elsewhere^10,11,13,14^.

### Measures

*Nicotine harm perceptions (outcome)*

Respondents were asked: ‘How harmful do you think nicotine is to health?’ and responded on a 5-point scale from ‘not at all’ to ‘extremely’ harmful. We dichotomized responses to measure two different types of misperceptions: 1) overestimating the harm of nicotine (very/extremely harmful)^15^; and 2) incorrectly understanding the harm of nicotine (not at all/very/extremely harmful)^17^. Dichotomizing nicotine perceptions is a common strategy and has shown expected associations in prior research^5,15,16^.

*Tobacco product use*

Adults reported their lifetime, past 30-day (P30D), and frequency of use for various tobacco products, including cigarettes and LCCs. Current established use of cigarettes was defined as lifetime use of ≥100 cigarettes and current use of cigarettes every day or some days. LCC use was defined as P30D use of either cigarillos or little filtered cigars. We created a three-level variable, categorizing respondents who use cigarettes and/or LCCs into one of the following three groups: 1) current use of cigarettes without LCCs; 2) current use of cigarettes and LCCs; and 3) current use of LCCs without cigarettes. We excluded people who only use LCCs as blunts. We also created a variable capturing current use of any nicotine or tobacco product other than cigarettes and LCCs (i.e. electronic nicotine products, traditional cigars, pipe, hookah, snus, or other smokeless tobacco).

*Demographic characteristics*

We examined the following demographic characteristics using standard PATH variables available in the RUF^12^: age; sex; sexual orientation; ethnicity and race; and education level.

### Statistical analysis

We conducted analyses using Stata (V16.0)^17^ survey data procedures. We calculated the weighted descriptive statistics and weighted percent of people who had misperceptions of nicotine harms for each study variable and ran weighted multivariable logistic regressions (two-tailed, significance at 0.05) to assess the association between tobacco use status and nicotine harm perceptions, controlling for demographics. We report adjusted odds ratios (AORs) and 95% confidence intervals (CIs). We derived standard errors using the balanced repeated replication method^18^ with Fay’s adjustment set to 0.3 to increase estimate stability^19^. For the main analyses, we measured use of LCCs as P30D use and cigarettes as current established use (defined above). In the Supplementary file Tables, we use two other definitions which did not change the conclusions of this study: 1) P30D use of LCCs, cigarettes, and other tobacco; or 2) current established use of LCCs, cigarettes, and other tobacco.

## RESULTS

### Weighted sample descriptives

The analytic sample for this study included people who reported current LCC use and/or current established cigarette use (n=5675). See the Supplementary file for the total sample size using other definitions of current use. Among the analytic sample, cigarette use was the most prevalent tobacco use behavior (81.5%; 95% CI: 80.2–82.8), followed by LCC use without cigarettes (9.7%; 95% CI: 8.7–10.7), and then dual use of LCCs and cigarettes (8.8%; 95% CI: 8.0–9.7); 69.5% (95% CI: 68.1–70.9) of people who reported current LCC and/or cigarette use did not use any other tobacco products; 60.4% (95% CI: 58.4–62.3) of people in this group were aged 25–54 years, 56.0% (95% CI: 54.2–57.7) male, 88.0% (95% CI: 87.0–88.9) straight, 63.4% (95% CI: 61.8–64.9) non-Hispanic (NH) White, and 31.1% (95% CI: 29.6–32.7) had graduated high school without any advanced education.

### Nicotine harm perceptions

Overall, people who use LCCs and/or cigarettes are likely to overestimate the harm of nicotine (63.0%; 95% CI: 61.8–64.2) and have incorrect perceptions about the harm of nicotine (65.7%; 95% CI: 64.6–66.9). See Supplementary file Table 1 for the breakdown of the percent overestimating the harm of nicotine and with incorrect harm perceptions of nicotine by each study variable.

### Association between tobacco use and nicotine harm perceptions

Table 1 shows the weighted AOR for each study variable’s association with overestimating the harm of nicotine and having incorrect harm perceptions about nicotine. Current use of LCCs and/or cigarettes was not significantly associated with either outcome. Other study variables were significantly associated with harm perception outcomes. Using tobacco products other than LCCs or cigarettes was significantly associated with lower odds of overestimating (AOR=0.70; 95% CI: 0.61–0.81) and being incorrect (AOR=0.73; 95% CI: 0.63–0.84) than not using other tobacco products with LCCs and/or cigarettes. Adults aged 25–54 years were more likely to overestimate (AOR=1.28; 95% CI: 1.03–1.59) and more likely to be incorrect (AOR=1.27; 95% CI: 1.03–1.57) compared to young adults aged 18–24 years. Males were less likely to overestimate (AOR=0.67; 95% CI: 0.57–0.78) and less likely to be incorrect (AOR=0.69; 95% CI: 0.59–0.81) compared to females. Both NH Blacks and Hispanics were more likely to overestimate (NH Black, AOR=1.28; 95% CI: 1.05–1.55; Hispanic, AOR=1.62; 95% CI: 1.31–2.00) and more likely to be incorrect (NH Black, AOR=1.39; 95% CI: 1.15–1.67; Hispanic, AOR=1.63; 95% CI: 1.32–2.02) about nicotine harm perceptions compared to NH Whites. Finally, those with a Bachelor’s degree or an advanced degree were less likely to overestimate (AOR=0.76; 95% CI: 0.58–0.99) and less likely to be incorrect (AOR=0.64; 95% CI: 0.48–0.86) about nicotine harm perceptions compared to those with lower than a high school education.

**Table 1:** Weighted (W7 Adult RUF with W7 Cohort 7 cross-sectional weights) association between current established cigarette^a^ use and P30D LCC use and nicotine harm perceptions^b^ at W7, United States, 2022–2023 (N=5507)

*Variables*	*AOR (95% CI)* *Overestimate of harm* *perception (vs no overestimate)*	*AOR (95% CI)* *Incorrect harm perception* *(vs correct)*
**Tobacco use status**		
Current established use of cigarettes without P30D use of LCCs ®	1	1
Current established use of cigarettes and P30D use of LCCs	1.05 (0.80–1.39)	1.16 (0.88–1.53)
P30D use of LCCs without current use of cigarettes	1.05 (0.82–1.34)	1.19 (0.93–1.53)
**Covariates**		
**P30D use of at least one other nicotine or tobacco product^c^**		
No ®	1	1
Yes	0.70*** (0.61–0.81)	0.73*** (0.63–0.84)
**Age** (years)		
Young adults (18–24) ®	1	1
Adults (25–54)	1.28* (1.03–1.59)	1.27* (1.03–1.57)
Older adults (≥55)	1.21 (0.94–1.56)	1.14 (0.88–1.47)
**Sex**		
Female ®	1	1
Male	0.67*** (0.57–0.78)	0.69*** (0.59–0.81)
**Sexual orientation**		
Straight ®	1	1
Lesbian, gay, bisexual, something else, not sure, don’t know	0.99 (0.80–1.23)	1.11 (0.89–1.38)
**Race/ethnicity**		
Non-Hispanic, White ®	1	1
Non-Hispanic, Black	1.28* (1.05–1.55)	1.39*** (1.15–1.67)
Non-Hispanic, other race, including multi-racial	1.29 (0.99–1.68)	1.28 (0.99–1.64)
Hispanic	1.62*** (1.31–2.00)	1.63*** (1.32–2.02)
**Education level**		
Lower than high school ®	1	1
GED	0.88 (0.67–1.14)	0.83 (0.61–1.12)
High school graduate	0.91 (0.71–1.16)	0.78 (0.58–1.04)
Some college (no degree) or associates	0.82 (0.65–1.02)	0.70** (0.55–0.90)
Bachelor’s degree or advanced degree	0.76* (0.58–0.99)	0.64** (0.48–0.86)
**Model F statistic** (p)^d^	**7.38 (<0.05)**	**7.38 (<0.05)**

a Current established use of cigarettes is defined as lifetime use of ≥100 cigarettes and current use of cigarettes every day or on some days.

b Respondents were asked: ‘How harmful do you think nicotine is to health?’ and responded on a 5-point scale (not at all harmful; slightly harmful; somewhat harmful; very harmful; extremely harmful). We dichotomized responses to measure two different types of misperceptions: 1) Whether the respondent overestimates the harm of nicotine: ‘0=not overestimate (not at all/ slightly/somewhat harmful)’ vs ‘1=overestimate (very/extremely harmful)’14; and 2) Whether the respondent incorrectly understands the harm of nicotine: ‘0=correct (slightly/ somewhat harmful)’ vs ‘1=incorrect (not at all/very/extremely harmful)’.

c Other tobacco products variable includes electronic nicotine products, traditional cigars, pipe, hookah, snus, and other smokeless tobacco.

d Significant F statistic shows model fit. W7: Wave 7. RUF: restricted use file. LCC: little cigars and cigarillos. P30D: past-30 day. GED: general educational development. AOR: adjusted odds ratio. ® Reference categories.

* p<0.05.

** p<0.01.

*** p<0.001.

## DISCUSSION

We examined nicotine harm misperceptions among people who use LCCs and found that a similar percentage of US adults who use LCCs overestimate or are generally incorrect about the harms of nicotine as those who use cigarettes, or dual use both products. We also found that US adults who reported the use of at least one additional tobacco product other than cigarettes and LCCs were significantly less likely to overestimate nicotine health harms and report incorrect nicotine perceptions than those who only used cigarettes and/or LCCs. Additionally, males (vs females), young adults (vs adults 25–54 years), NH White people (vs NH Black and NH Other), and those who reported education level beyond high school (vs those with lower than a high school education) were significantly less likely to overestimate nicotine health harms and hold incorrect nicotine perceptions.

Prior research indicates that individuals in population sub-groups that overestimate the harms of nicotine may not realize that nicotine replacement therapy is an FDA-approved, safe, and effective cessation tool^4,5^. Ongoing monitoring of nicotine misperceptions in these specific groups of LCC users will help detect changes to the absolute prevalence of misperceptions following regulatory actions and estimate how these misperceptions impact the continued use of combustible tobacco products.

## Strengths and limitations

This research has strengths, including that it examined adult LCC users from the US who are not well represented in tobacco research, and that the research uses the most recent nationally representative PATH Study data to provide an estimate of nicotine perceptions.

This research has, however, some limitations including that it uses self-reported data susceptible to social desirability biases, that it is cross-sectional and thus does not allow for causal inference, that it is subject to residual confounding, and that it was conducted using only US data, which limits its generalizability for adult LCC users in other countries.

## CONCLUSIONS

Our analyses noted that people who use LCCs are equally likely to overestimate or be incorrect about nicotine harms as those who exclusively or dual use cigarettes, but using additional products is associated with correct responses about nicotine harms. Future research could explore nicotine misperceptions among other populations and examine how demographic variables such as age and sex interact with tobacco use status in predicting nicotine misperceptions.

## Supplementary Material



## Data Availability

The data supporting this research are available from the following source: https://www.icpsr.umich.edu/web/NAHDAP/studies/36231
