# Supplementary material for: Perceptions of nicotine harm among adults who use little cigars and cigarillos: A cross-sectional analysis of wave 7 of the Population Assessment of Tobacco and Health (PATH) Study 2022–2023
Source: Tob Induc Dis. 2026 Jan 16;24:10.18332/tid/214722. doi: 10.18332/tid/214722 (PMC12810323; doi:10.18332/tid/214722)
Supplement: Supplementary file 1 [file TID-24-03-s1.pdf]

Supplementary Table 1: Weighted (W7 Adult RUF with W7 Cohort 7 Cross-sectional Weights; United States, 2022-2023) descriptive statistics of the study sample at W7 and weighted proportion who overestimate or incorrectly report nicotine harm[1] (Total N = Current Established Cigarette[2] and P30D LCC Users = 5,675)

	Total N and sample percentage (CI)		Percentage (CI) overestimating the harm of nicotine	Percentage (CI) incorrect about the harm of nicotine
**Overall**	N/A		63.0% (61.8-64.2)	65.7% (64.6-66.9)
Cur Estd Use of Cigarettes and P30D Use of LCCs				
Cur estd use of cigarettes without P30D use of LCCs	4,466	81.5% (80.2-82.8)	63.5% (62.1-64.8)	65.6% (64.2-66.9)
Cur estd use of cigarettes and P30D use of LCCs	539	8.8% (8.0-9.7)	61.5% (55.5-67.3)	67.0% (61.2-72.3)
P30D use of LCCs without cur estd use of cigarettes	670	9.7% (8.7-10.7)	60.1% (54.8-65.2)	65.9% (60.8-70.7)
P30D Other Tobacco Use				
P30D use of at least one other nicotine or tobacco product[3]				
No	3,725	69.5% (68.1-70.9)	65.6% (64.0-67.2)	67.7% (66.2-69.2)
Yes	1,897	30.5% (29.1-31.9)	57.0% (54.4-59.6)	61.3% (58.8-63.7)
Sociodemographic Variables				
Age (years)				
Young adults (18-24)	661	5.6% (5.1-6.1)	55.2% (50.7-59.7)	60.7% (56.1-65.2)
Adults (25-54)	3,263	60.4% (58.4-62.3)	63.2% (61.3-65.1)	66.4% (64.6-68.2)
Older adults (55+)	1,751	34.1% (32.1-36.1)	63.8% (61.3-66.2)	65.4% (62.8-67.8)
Sex				
Female	2,891	44.0% (42.3-45.8)	67.8% (65.7-69.8)	69.8% (67.7-71.8)
Male	2,784	56.0% (54.2-57.7)	59.2% (57.0-61.3)	62.6% (60.5-64.6)
Sexual Orientation				
Straight	4,752	88.0% (87.0-88.9)	62.8% (61.5-64.1)	65.3% (64.0-66.5)
Lesbian, gay, bisexual, something else, not sure, don't know	840	12.0% (11.1-13.0)	63.9% (59.6-67.9)	68.9% (64.8-72.6)
Race/Ethnicity				
Non-Hispanic, White	3,290	63.4% (61.8-64.9)	60.3% (58.7-61.9)	62.6% (61.1-64.2)
Non-Hispanic, Black	1,153	15.4% (14.4-16.5)	65.8% (62.4-69.1)	70.5% (67.4-73.4)
Non-Hispanic, Other race including multi-racial	397	8.1% (7.1-9.2)	65.2% (59.7-70.3)	67.4% (62.3-72.1)
Hispanic	835	13.2% (12.1-14.3)	71.2% (67.2-74.9)	74.1% (70.4-77.5)
Educational Attainment				
Less than high school	964	15.6% (14.6-16.6)	66.3% (62.3-70.1)	71.4% (67.2-75.3)
GED	645	11.5% (10.5-12.7)	62.0% (57.1-66.7)	66.2% (61.1-71.0)
High school graduate	1,501	31.1% (29.6-32.7)	63.6% (60.7-66.4)	65.5% (62.5-68.4)
Some college (no degree) or associates	1,878	29.9% (28.5-31.4)	61.8% (59.5-64.0)	64.0% (61.7-66.2)
Bachelor's degree or advanced degree	653	11.8% (10.8-13.0)	60.4% (56.0-64.6)	62.5% (58.0-66.6)

Abbreviations: W7=Wave 7; RUF=restricted use file; LCC=little cigars and cigarillos; P30D=past-30 day; CI=confidence interval; Cur Estd=current established; N/A=not applicable; GED=General Educational Development

[1] Respondents were asked "How harmful do you think nicotine is to health?" and responded on a 5-point scale (Not at All Harmful; Slightly Harmful; Somewhat Harmful; Very Harmful; Extremely Harmful). We dichotomized responses to measure two different types of misperceptions: 1) Whether the respondent overestimates the harm of nicotine: "0=Not Overestimate (Not At All/Slightly/Somewhat Harmful)" vs. "1=Overestimate (Very/Extremely Harmful)"14; and 2) Whether the respondent incorrectly understands the harm of nicotine: "0=Correct (Slightly/Somewhat Harmful)" vs. "1= Incorrect (Not at all/Very/Extremely Harmful)".

[2] Current established use of cigarettes is defined as lifetime use of ≥100 cigarettes and current use of cigarettes every day or on some days.

[3] Other tobacco products variable includes electronic nicotine products, traditional cigars, pipe, hookah, snus, and other smokeless tobacco.

Supplemental Table 2: Weighted (W7 Adult RUF with W7 Cohort 7 Cross-sectional Weights; United States, 2022-2023) descriptive statistics of the study sample at W7 and weighted proportion who overestimate or incorrectly report nicotine harm[1] (Total N = P30D LCC and Cigarette Users = 6,619)

	Total N and sample percentage (CI)		Percentage (CI) overestimating the harm of nicotine	Percentage (CI) incorrect about the harm of nicotine
**Overall**	N/A		64.2% (63.0-65.4)	67.2% (66.1-68.2)
P30D Use of Cigarettes and LCCs				
P30D use of cigarettes without LCCs	5,409	83.9% (82.7-85.0)	64.9% (63.5-66.1)	67.3% (66.1-68.6)
P30D use of cigarettes and LCCs	720	9.9% (9.1-10.8)	61.6% (56.6-66.5)	66.9% (62.1-71.5)
P30D use of LCCs without cigarettes	490	6.2% (5.5-7.0)	59.5% (52.9-65.8)	65.7% (59.4-71.5)
P30D Other Tobacco Use				
P30D use of at least one other nicotine or tobacco product[2]				
No	4,191	67.7% (66.4-69.0)	67.0% (65.5-68.4)	69.3% (67.9-70.7)
Yes	2,428	32.3% (31.0-33.6)	58.4% (56.0-60.8)	62.7% (60.5-64.9)
Sociodemographic variables				
Age (years)				
Young adults (18-24)	1,084	7.9% (7.3-8.5)	59.5% (56.3-62.7)	64.7% (61.6-67.7)
Adults (25-54)	3,671	59.6% (57.7-61.4)	64.0% (62.0-65.9)	67.3% (65.9-69.2)
Older adults (55+)	1,864	32.6% (30.7-34.4)	65.8% (63.4-68.1)	67.5% (65.1-69.8)
Sex				
Female	3,367	44.4% (42.7-46.1)	69.7% (67.9-71.5)	71.8% (70.0-73.5)
Male	3,252	55.6% (53.9-57.3)	59.8% (57.7-61.8)	63.5% (61.6-65.5)
Sexual Orientation				
Straight	5,448	87.2% (86.3-88.1)	63.9% (62.6-65.1)	66.6% (65.4-67.7)
Lesbian, gay, bisexual, something else, not sure, don't know	1,072	12.8% (11.9-13.7)	66.3% (62.7-69.8)	71.5% (68.1-74.7)
Race/Ethnicity				
Non-Hispanic, White	3,733	62.1% (60.6-63.7)	61.7% (60.2-63.2)	64.1% (62.6-65.6)
Non-Hispanic, Black	1,286	15.1% (14.0-16.2)	66.3% (63.1-69.4)	71.2% (68.2-74.0)
Non-Hispanic, Other race including multi-racial	483	8.4% (7.5-9.5)	66.1% (60.8-71.0)	69.1% (64.6-73.4)
Hispanic	1,117	14.4% (13.3-15.6)	71.7% (68.5-74.7)	75.2% (72.4-77.7)
Educational Attainment				
Less than high school	1,082	15.1% (14.1-16.1)	66.4% (62.6-70.0)	72.5% (68.5-76.1)
GED	700	10.8% (9.8-11.9)	62.4% (57.5-66.9)	66.5% (61.6-71.1)
High school graduate	1,763	30.4% (28.9-32.0)	63.7% (60.9-66.5)	65.9% (63.1-68.7)
Some college (no degree) or associates	2,193	29.9% (28.6-31.2)	63.7% (61.5-65.9)	66.0% (63.8-67.2)
Bachelor's degree or advanced degree	844	13.8% (12.8-14.9)	65.1% (61.2-68.8)	67.0% (63.1-70.7)

Abbreviations: W7=Wave 7; RUF=restricted use file; LCC=little cigars and cigarillos; P30D=past-30 day; CI=confidence interval; Cur Estd=current established; N/A=not applicable; GED=General Educational Development

[1] Respondents were asked "How harmful do you think nicotine is to health?" and responded on a 5-point scale (Not at All Harmful; Slightly Harmful; Somewhat Harmful; Very Harmful; Extremely Harmful). We dichotomized responses to measure two different types of misperceptions: 1) Whether the respondent overestimates the harm of nicotine: "0=Not Overestimate (Not At All/Slightly/Somewhat Harmful)" vs. "1=Overestimate (Very/Extremely Harmful)"14; and 2) Whether the respondent incorrectly understands the harm of nicotine: "0=Correct (Slightly/Somewhat Harmful)" vs. "1= Incorrect (Not at all/Very/Extremely Harmful)".

[2] Other tobacco products variable includes electronic nicotine products, traditional cigars, pipe, hookah, snus, and other smokeless tobacco.

Supplemental Table 3: Weighted (W7 Adult RUF with W7 Cohort 7 Cross-sectional Weights; United States, 2022-2023) descriptive statistics of the study sample at W7 and weighted proportion who overestimate or incorrectly report nicotine harm[1] (Total N = Current Established[2] LCC and Cigarette Users = 5,435)

	Total N and sample percentage (CI)		Percentage (CI) overestimating the harm of nicotine	Percentage (CI) incorrect about the harm of nicotine
**Overall**	N/A		63.1% (61.8-64.3)	65.6% (64.5-66.8)
Current Established Use of Cigarettes and LCCs				
Cur estd use of cigarettes without LCCs	4,818	89.8% (88.7-90.7)	63.6% (62.2-64.9)	66.0% (64.7-67.2)
Cur estd use of cigarettes and LCCs	311	5.3% (4.7-5.9)	61.6% (53.0-69.5)	64.9% (56.3-72.7)
Cur estd use of LCCs without cigarettes	306	5.0% (4.3-5.7)	56.6% (48.9-63.9)	60.9% (53.2-68.2)
Current Established Other Tobacco Use				
Cur estd use of at least one other nicotine or tobacco product[3]				
No	4,223	80.4% (79.3-81.5)	65.1% (63.6-66.5)	67.3% (65.9-68.7)
Yes	1,212	19.6% (18.5-20.7)	55.0% (51.6-58.3)	58.9% (55.5-62.2)
Sociodemographic variables				
Age (years)				
Young adults (18-24)	502	4.5% (4.1-5.0)	54.3% (49.6-58.9)	59.8% (54.8-64.5)
Adults (25-54)	3,199	60.8% (58.8-62.8)	63.3% (61.3-65.2)	66.2% (64.3-68.0)
Older adults (55+)	1,734	34.6% (32.7-36.7)	64.0% (61.4-66.5)	65.5% (62.9-68.0)
Sex				
Female	2,841	45.0% (43.2-46.9)	67.8% (65.7-69.9)	69.7% (67.6-71.7)
Male	2,594	55.0% (53.2-56.8)	59.2% (57.0-61.4)	62.3% (60.2-64.4)
Sexual Orientation				
Straight	4,557	88.1% (87.1-89.0)	62.8% (61.4-64.2)	65.2% (63.9-66.4)
Lesbian, gay, bisexual, something else, not sure, don't know	802	11.9% (11.0-12.9)	64.6% (60.3-68.8)	68.8% (64.7-72.7)
Race/Ethnicity				
Non-Hispanic, White	3,192	63.9% (62.3-65.4)	60.5% (58.9-62.1)	62.8% (61.3-64.3)
Non-Hispanic, Black	1,081	15.1% (14.0-16.2)	66.0% (62.2-69.6)	70.4% (66.9-73.8)
Non-Hispanic, Other race including multi-racial	382	8.1% (7.1-9.2)	64.7% (58.9-70.1)	66.7% (61.3-71.7)
Hispanic	780	12.9% (11.9-14.1)	71.3% (67.2-75.0)	73.3% (69.4-76.9)
Educational Attainment				
Less than high school	949	15.9% (14.8-17.0)	66.5% (62.6-70.2)	71.2% (67.0-75.0)
GED	629	11.7% (10.6-12.9)	62.8% (57.8-67.5)	66.8% (61.7-71.6)
High school graduate	1,414	31.1% (29.6-32.7)	63.3% (60.3-66.3)	65.1% (61.9-68.2)
Some college (no degree) or associates	1,804	30.0% (28.6-31.5)	62.6% (60.3-65.0)	64.6% (62.3-66.9)
Bachelor's degree or advanced degree	608	11.2% (10.2-12.4)	59.2% (54.6-63.7)	60.9% (56.3-65.3)

Abbreviations: W7=Wave 7; RUF=restricted use file; LCC=little cigars and cigarillos; P30D=past-30 day; CI=confidence interval; Cur Estd=current established; N/A=not applicable; GED=General Educational Development

[1] Respondents were asked "How harmful do you think nicotine is to health?" and responded on a 5-point scale (Not at All Harmful; Slightly Harmful; Somewhat Harmful; Very Harmful; Extremely Harmful). We dichotomized responses to measure two different types of misperceptions: 1) Whether the respondent overestimates the harm of nicotine: "0=Not Overestimate (Not At All/Slightly/Somewhat Harmful)" vs. "1=Overestimate (Very/Extremely Harmful)"14; and 2) Whether the respondent incorrectly understands the harm of nicotine: "0=Correct (Slightly/Somewhat Harmful)" vs. "1= Incorrect (Not at all/Very/Extremely Harmful)".

[2] Current established use of cigarettes is defined as lifetime use of ≥100 cigarettes and current use of cigarettes every day or on some days. Current established use for other products, including cigars, is defined as "fairly regular" use and current use every day or on some days.

[3] Other tobacco products variable includes electronic nicotine products, traditional cigars, pipe, hookah, snus, and other smokeless tobacco.

Supplemental Table 4: Weighted (W7 Adult RUF with W7 Cohort 7 Cross-sectional Weights; United States, 2022-2023) association between P30D tobacco use and nicotine harm perceptions[1] at W7 (N=6,482)

	**Adjusted Odds Ratio (CI) Overestimate of Harm Perception (v. No Overestimate)**	**Adjusted Odds Ratio (CI) Incorrect Harm Perception (v. Correct)**
P30D tobacco use categories		
P30D use of cigarettes without LCCs	Ref	Ref
P30D use of cigarettes and LCCs	1.00 (0.79-1.28)	1.07 (0.84-1.36)
P30D use of LCCs without cigarettes	0.92 (0.69-1.23)	1.05 (0.78-1.40)
Covariates		
P30D use of at least one other nicotine or tobacco product[2]		
No	Ref	Ref
Yes	**0.71*** (0.62-0.81)**	**0.74*** (0.65-0.84)**
Age (years)		
Young adults (18-24)	Ref	Ref
Adults (25-54)	1.07 (0.91-1.26)	1.04 (0.89-1.23)
Older adults (55+)	1.07 (0.87-1.33)	0.99 (0.80-1.22)
Sex		
Female	Ref	Ref
Male	**0.64*** (0.55-0.73)**	**0.67*** (0.58-0.77)**
Sexual Orientation		
Straight	Ref	Ref
Lesbian, gay, bisexual, something else, not sure, don't know	1.03 (0.85-1.25)	1.16 (0.95-1.41)
Race/ethnicity		
Non-Hispanic, White	Ref	Ref
Non-Hispanic, Black	**1.26* (1.06-1.51)**	**1.39*** (1.16-1.66)**
Non-Hispanic, Other race including multi-racial	1.21 (0.93-1.5)	1.24 (0.98-1.57)
Hispanic	**1.63*** (1.38-1.94)**	**1.69*** (1.44-1.99)**
Educational attainment		
Less than high school	Ref	Ref
GED	0.90 (0.69-1.16)	0.82 (0.61-1.09)
High school graduate	0.93 (0.74-1.17)	0.77 (0.59-1.01)
Some college (no degree) or associates	0.90 (0.73-1.11)	**0.74* (0.59-0.94)**
Bachelor's degree or advanced degree	0.98 (0.77-1.26)	0.80 (0.61-1.05)
**Model F statistic (p-value)[3]**	**9.37 (p<0.05)**	**9.61 (p<0.05)**

Abbreviations: W7=Wave 7; RUF=restricted use file; LCC=little cigars and cigarillos; P30D=past-30 day; CI=confidence interval; Cur Estd=current established; Ref=reference group; GED=General Educational Development

[1] Respondents were asked "How harmful do you think nicotine is to health?" and responded on a 5-point scale (Not at All Harmful; Slightly Harmful; Somewhat Harmful; Very Harmful; Extremely Harmful). We dichotomized responses to measure two different types of misperceptions: 1) Whether the respondent overestimates the harm of nicotine: "0=Not Overestimate (Not At All/Slightly/Somewhat Harmful)" vs. "1=Overestimate (Very/Extremely Harmful)"14; and 2) Whether the respondent incorrectly understands the harm of nicotine: "0=Correct (Slightly/Somewhat Harmful)" vs. "1= Incorrect (Not at all/Very/Extremely Harmful)".
[2] Other tobacco products variable includes electronic nicotine products, traditional cigars, pipe, hookah, snus, and other smokeless tobacco.
[3] Significant F statistic shows model fit.
* p<0.05, ** p<0.01, *** p<0.001

Supplemental Table 5: Weighted (W7 Adult RUF with W7 Cohort 7 Cross-sectional Weights; United States, 2022-2023) association between current established tobacco use[1] and nicotine harm perceptions[2] at W7 (N=5,326)

	**Adjusted Odds Ratio (CI) Overestimate of Harm Perception (v. No Overestimate)**	**Adjusted Odds Ratio (CI) Incorrect Harm Perception (v. Correct)**
Current established tobacco use categories		
Current established use of cigarettes without LCCs	Ref	Ref
Current established use of cigarettes and LCCs	0.98 (0.68-1.43)	0.98 (0.66-1.45)
Current established use of LCCs without cigarettes	0.90 (0.63-1.27)	0.94 (0.66-1.35)
Covariates		
Current established use of at least one other nicotine or tobacco product[3]		
No	Ref	Ref
Yes	**0.69*** (0.59-0.81)**	**0.72*** (0.61-0.86)**
Age (years)		
Young adults (18-24)	Ref	Ref
Adults (25-54)	**1.31* (1.04-1.64)**	1.24 (0.98-1.56)
Older adults (55+)	1.28 (0.97-1.67)	1.15 (0.87-1.51)
Sex		
Female	Ref	Ref
Male	**0.67*** (0.57-0.79)**	**0.70*** (0.59-0.82)**
Sexual Orientation		
Straight	Ref	Ref
Lesbian, gay, bisexual, something else, not sure, don't know	1.05 (0.84-1.31)	1.13 (0.90-1.42)
Race/Ethnicity		
Non-Hispanic, White	Ref	Ref
Non-Hispanic, Black	**1.29* (1.05-1.59)**	**1.42** (1.16-1.75)**
Non-Hispanic, Other race including multi-racial	1.27 (0.98-1.64)	1.24 (0.97-1.59)
Hispanic	**1.60*** (1.31-1.95)**	**1.57*** (1.28-1.92)**
Educational Attainment		
Less than high school	Ref	Ref
GED	0.88 (0.67-1.16)	0.85 (0.63-1.15)
High school graduate	0.90 (0.71-1.16)	0.78 (0.59-1.04)
Some college (no degree) or associates	0.85 (0.69-1.06)	**0.74* (0.59-0.94)**
Bachelor's degree or advanced degree	**0.74* (0.57-0.95)**	**0.63** (0.48-0.83)**
**Model F statistic (p-value)[4]**	**7.18 (p<0.05)**	**6.09 (p<0.05)**

Abbreviations: W7=Wave 7; RUF=restricted use file; LCC=little cigars and cigarillos; P30D=past-30 day; CI=confidence interval; Cur Estd=current established; Ref=reference group; GED=General Educational Development

[1] Current established use of cigarettes is defined as lifetime use of ≥100 cigarettes and current use of cigarettes every day or on some days. Current established use for other products, including cigars, is defined as "fairly regular" use and current use every day or on some days.
[2] Respondents were asked "How harmful do you think nicotine is to health?" and responded on a 5-point scale (Not at All Harmful; Slightly Harmful; Somewhat Harmful; Very Harmful; Extremely Harmful). We dichotomized responses to measure two different types of misperceptions: 1) Whether the respondent overestimates the harm of nicotine: "0=Not Overestimate (Not At All/Slightly/Somewhat Harmful)" vs. "1=Overestimate (Very/Extremely Harmful)"14; and 2) Whether the respondent incorrectly understands the harm of nicotine: "0=Correct (Slightly/Somewhat Harmful)" vs. "1= Incorrect (Not at all/Very/Extremely Harmful)".
[3] Other tobacco products variable includes electronic nicotine products, traditional cigars, pipe, hookah, snus, and other smokeless tobacco.
[4] Significant F statistic shows model fit.
* p<0.05, ** p<0.01, *** p<0.001

© **2026 Fidalgo A. et al.**
